# Case Report: A novel mutation in *TNFAIP3* in a patient with type 1 diabetes mellitus and haploinsufficiency of A20

**DOI:** 10.3389/fendo.2023.1131437

**Published:** 2023-05-31

**Authors:** Conghui Cao, Xue Fu, Xiaoli Wang

**Affiliations:** Department of Endocrinology and Metabolism, Institute of Endocrinology, National Health Commission (NHC) Key Laboratory of Diagnosis and Treatment of Thyroid Diseases, The First Hospital of China Medical University, Shenyang, China

**Keywords:** *TNFAIP3*, type 1 diabetes mellitus, HA20, mutation, case report

## Abstract

**Background:**

Haploinsufficiency of A20 (HA20) is a monogenic autosomal-dominant genetic autoinflammatory disease caused by loss of function mutations in the *TNFAIP3* gene. The predominant autoimmune phenotype associated with HA20 varies significantly, presenting with fever, recurrent oral and genital ulcers, skin rash, gastrointestinal and musculoskeletal symptoms, and other clinical manifestations, all of which indicate an early-onset of autoinflammatory disorder. Genetic linkage between TNFAIP3 and T1DM was reported in GWAS studies. However, only a few cases of HA20 combined with T1DM have been reported.

**Case description:**

A 39-year-old man with a history of type 1 diabetes mellitus since 19 years was admitted to the Department of Endocrinology and Metabolism, First Affiliated Hospital of China Medical University. He also suffered from recurring and minor mouth ulcers since early childhood. His laboratory evaluation results revealed reduced islet function, normal lipid profile, HbA1c of 7%, elevated glutamate decarboxylase antibodies, elevated hepatic transaminases, and elevated thyroid-related antibodies with normal thyroid function. Notably, the patient was diagnosed in adolescence and never had ketoacidosis, the islets were functioning despite the long disease duration, his abnormal liver function could not be reasonably explained, and he had early onset Behcet’s-like disease symptom. Hence, although he was on routine follow-up for diabetes, we communicated with him and obtained consent for genetic testing. Whole-exome sequencing revealed a novel c.1467_1468delinsAT heterozygous mutation in the gene TNFAIP3, which is located in exon 7, resulting in a stop-gained type mutation p.Q490*. With good but mild fluctuating glycemic control, the patient received intensive insulin therapy with long-acting and short-acting insulin. The liver function was improved by using ursodeoxycholic acid 0.75 mg/d during the follow-up.

**Conclusion:**

We report a novel pathogenic mutation in *TNFAIP3* that results in HA20 in a patient with T1DM. In addition, we analyzed the clinical feathers of such patients and summarized the cases of five patients with HA20 co-presented with T1DM. When T1DM co-occurs with autoimmune diseases or other clinical manifestations, such as oral and/or genital ulcers and chronic liver damage, the possibility of an HA20 must be considered. Early and definitive diagnosis of HA20 in such patients may inhibit the progression of late-onset autoimmune diseases, including T1DM.

## 1 Introduction

Monogenic conditions are known to cause autoimmune diabetes that is clinically indistinguishable from T1DM. To date, nine disease-causing genes have been reported, including *AIRE*, *CTLA4*, *FOXP3*, *IL2RA*, *ITCH*, *LRBA*, *SIRT1*, *STAT1*, and *STAT3* (1–3). Compared with T1DM, autoimmune monogenic diabetes typically manifests earlier and is more likely to be associated with multiple autoimmune diseases, such as immune dysregulation, polyendocrinopathy, enteropathy, and X-linked (IPEX) syndrome, which is caused by mutations in the FOXP3 and AIRE genes (2).

The tumor necrosis factor alpha–induced protein 3 (TNFAIP3) gene encodes the ubiquitin-editing enzyme A20, which acts as a negative regulator of the nuclear factor-κB (NF-κB) pathway (4). Haploinsufficiency of A20 (HA20) is a monogenic autosomal-dominant genetic autoinflammatory disease, which was first described and named by Zhou et al. in 2016 (5). The predominant autoimmune phenotype associated with HA20 varies significantly in reported cases, presenting with fever, recurrent oral and genital ulcers, skin rash, gastrointestinal and musculoskeletal symptoms, and other clinical manifestations, which are all indicating an early-onset of autoinflammatory disorder (6). Genetic linkage between *TNFAIP3* gene and T1DM was also reported in GWAS studies (7–9).

Although more than 100 cases of HA20 combined with multiple autoimmune disorders have been reported in the relevant literature, there are only a few reports of combined T1DM and their clinical feathers. Here we report the case of a patient with HA20 who initially presented with T1DM. The patient was diagnosed in adolescence and never had ketoacidosis, his islet function was still not completely failed after such a long time (fasting C-peptide: >200 pmol/L), his abnormal liver function could not be reasonably explained, and he had early onset Behcet’s-like disease symptom. Hence, although he was only on routine follow-up for diabetes, we communicated with him and obtained his consent for genetic testing. A new nonsense mutation in *TNFAIP3* was identified and the patient was diagnosed with HA20. At present, there is no recommended treatment plan for HA20 combined with T1DM; therefore, we did not change his treatment plan and continued to use insulin to control blood glucose. Here we report the clinical characteristics of our patient and present the review cases of HA20 combined with T1DM based on the relevant literature.

## 2 Case report

A 39-year-old male patient with hyperglycemia for 20 years was admitted to the Department of Endocrinology and Metabolism, First Affiliated Hospital of China Medical University. He was diagnosed with type 1 diabetes mellitus at the age of 19 years and has been on insulin therapy since then. He also had recurring and minor oral and genital ulcers since early childhood. His parents are healthy and nonconsanguineous. He is a married man with no children. The physical examination revealed the following characteristics: height, 170.0 cm; weight, 65.0 kg; body mass index, 22.5 kg/m^2^; and vision and hearing were normal.

As shown in **Table 1**, laboratory examination results revealed reduced islet function, a normal lipid profile, an HbA1c of 7%, increased glutamate decarboxylase (GAD) antibodies, elevated liver transaminases, and increased thyroid-related antibodies with normal thyroid function. Electromyography, cardiac and vascular color Doppler ultrasound, and abdominal color Doppler ultrasound were normal.

**Table 1:** Laboratory investigations.

Test	At diagnosis					Follow up (5 month)	Normal values
	Fasting	30 min after OGTT	60 min after OGTT	120 min after OGTT	180 min after OGTT	Fasting	
PG (mmol/L)	7.25	11.45	17.34	20.45	19.36	6.95	–
INS (mIU/L)	6.99	7.28	7.95	11.98	12.77	–	–
CP (pmol/L)	319.7	371.7	504.7	549.6	695.5	–	–
HbA1c (%)	7					6.8	4.4–6
LDL-c (mmol/L)	2.32					2.34	0–3.64
TC (mmol/L)	4.58					4.35	0–5.72
TG (mmol/L)	1.08					0.59	0–1.7
HDL-c (mmol/L)	1.79					1.73	0.91–1.92
UA (µmol/L)	378					–	208–428
TSH (mIU/L)	2.93					3.96	0.35–4.94
fT4 (pmol/L)	12.06					12.56	9.01–19.05
fT3 (pmol/L)	3.82					4.12	2.63–5.7
TPOAb (IU/mL)	204.52					–	0–5.61
TGAb (IU/mL)	235.69					–	0–4.11
Anti-GAD antibody (IU/mL)	22.39					–	0–17
IAA (IU/mL)	11.43					–	0.41–20
ICA (IU/mL)	4.11					–	0–20
AST (U/L)	47					24	15–40
ALT (U/L)	89					29	9–50
GGT (U/L)	240					84	10–60
ALP (U/L)	224					180	45–125
RF (IU/mL)	6.3					–	0–14
CRP (mg/L)	5.8					–	0–6
Hemoglobin (g/L)	149					–	130-175
Leucocytes (10^9^/L)	4.03					–	3.50-9.50
Neutrophils (10^9^/L)	2.22					–	1.80-6.30
Lymphocytes (10^9^/L)	1.41					–	1.10-3.20
Monocytes (10^9^/L)	0.32					–	0.10-0.60
Platelets (10^9^/L)	195					–	125-350
Urine ketone	–					–	(-)

PG, plasma glucose; INS, serum insulin; CP, serum C-peptide; HbA1c, hemoglobin A1c; LDL-c, low density lipoprotein cholesterol; TC, total cholesterol; TG, triglyceride; HDL-c, high density lipoprotein cholesterol; UA, uric acid; TSH, thyrotropin-releasing hormone; fT4, free thyroxine; fT3, free triiodothyronine; TPOAb, anti-thyroid peroxidase antibody; TGAb, anti-thyroglobulin antibody; GAD, glutamic acid decarboxylase; IAA, insulin autoantibody; ICA, islet cell antibody; AST, aspartate transaminase; ALT, alanine amino transferase; GGT, gamma glutamyl transferase; ALP, alkaline phosphatase; RF, rheumatoid factor; CRP, C-reactive protein;

### 2.1 Genetic testing

**Figure 1:**
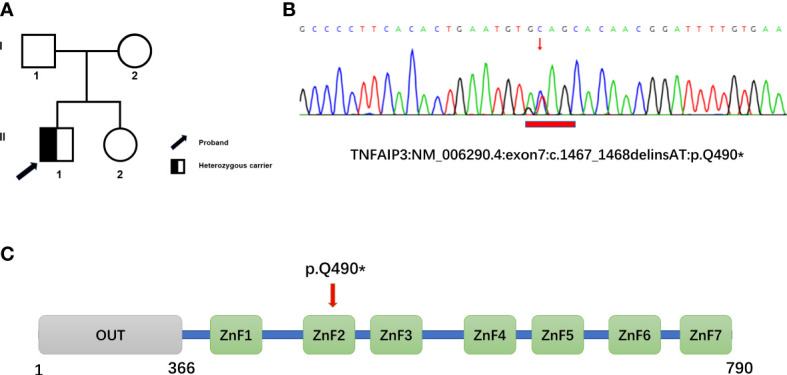
Family pedigree and genetic analyses. **(A)** Pedigree of the family with heterozygous mutation in *TNFAIP3*. **(B)** Whole-exome sequencing revealed a novel heterozygous mutation, c.1467_1468delinsAT (p.Glu490*), in *TNFAIP3* in the patient, which was confirmed by Sanger Sequencing. **(C)** Schematic diagram of A20 protein and the location of the new identified mutation. OUT, ovarian tumor domain; ZnF 1-7, zinc finger domains.

Genetic testing was performed using whole-exome high-throughput sequencing technology, and the data were analyzed using the Verita Trekker® Variant Site Detection System and Enliven® Variant Site Annotation Interpretation System developed by Berry Genetics. As a result, a c.1467_1468delinsAT heterozygous mutation in the gene TNFAIP3, located in exon 7, was discovered, resulting in a stop-gained type mutation, p.Q490* (**Figure 1**). The patient’s parents and sister were found to be devoid of this variant.

### 2.2 Treatment and follow-up

With good but mild fluctuating glycemic control, the patient received intensive insulin therapy with long-acting and short-acting insulin. The liver function was improved by using ursodeoxycholic acid 0.75 mg/d during the follow-up.

## 3 Discussion

HA20 is an autosomal-dominant genetic disease in which the NF-kB signaling pathway is less inhibited because of insufficient A20 production. The most common manifestation of HA20 is a Behcet’s-like disease symptom, which causes increased expression of proinflammatory cytokines mediated by the nuclear factor-κB pathway owing to A20-incuded weakened negative regulation of the nuclear factor-κB pathway. Further, the main manifestations of HA20 include oral and/or genital ulcers, fever, skin and musculoskeletal involvements, and gastrointestinal symptoms. No standard treatment has been established for the disease, and the treatment method is generally based on the dominant clinical phenotypes (6, 8, 9). Nearly half of the patients were reported to respond well to colchicine, with or without immunosuppressive or biological agents (6). However, even when the pathogenic variants are the same, patients’ clinical presentations and responses to treatment are highly heterogeneous (6).

TNFAIP3/A20 has been identified as the most upregulated anti-apoptotic gene in pancreatic islet beta cells (10) and prevents islet graft rejection in animal transplantation models (11). By increasing the threshold of inflammatory signaling, therapeutic administration of A20 promotes immune tolerance and survival of transplanted islets (11). In the absence of adequate A20 expression, soluble secretory factors released by cells may interfere with the expression of genes required for normal β-cell function (12).

We searched the literature and found 117 cases (in 60 families) of genetically confirmed HA20 (only cases with clinical information were counted), with 62 pathogenic variants in *TNFAIP3*. However, the number of cases of coexisting HA20 and T1DM remain small, with five cases (5/118, 4.2%) being added to our case (13–15). They almost always have oral and/or genital ulcers (4/5), autoimmune thyroid disease (AITD) (3/5), and other autoimmune diseases; moreover, they are particularly susceptible to hepatic cytolysis (3/5), which is generally uncommon in T1DM and APS2 (**Table 2**). Notably, these five patients did not present with other clinical manifestations of HA20, including early onset IBD, muscle-skeletal disorders with arthralgia and arthritis, and recurrent fever.

**Table 2:** Clinical feathers of patients with T1DM and HA20.

	P1	P2	P3	P4	P5
Age at the appearance of initial symptoms	10	Early childhood (<2 years)	childhood	16	5
Age at the onset of diabetes	10	9	NM	NM	19
Sex	Male	Female	Female	Female	Male
Aphthous ulcers	NM	yes	yes	yes	yes
Genital ulcers	NM	yes	yes	yes	yes
Arthralgia	NM	yes	no	no	yes
Hepatic cytolysis	yes	no	no	yes	yes
Hashimoto thyroiditis	no	yes	no	yes	yes
Other autoinflammatory diseases	cytopaenias, enteropathy and interstitial lung disease	NM	no	no	no
Anti-GAD antibody (IU/mL)	>2000	NM	NM	NM	22.39
Islet cell antibody	Detected	NM	NM	NM	Negative
pANCA	Detected	NM	NM	NM	NM
ANA	NM	NM	1/1280	1/1280	NM
TG	NM	NM	0.55	0.97	1.08
LDL-c	NM	NM	1.89	2.31	2.32
Variation	c.1466_1467delTG, p.V489Afs*7	c.1727dupC, p.His577Alafs*95	c.259C>T, p.Arg87*	c.259C>T, p.Arg87*	c.1467_1468delinsAT, p.Q490*
Domain	ZnF 2	between the ZnF 3 and 4	OUT	OUT	ZnF 2
Reference	[13]	[14]	[14]	[15]	This study

ZnF, zinc finger; OUT, ovarian tumor; NM, not mentioned.

The majority of the pathogenic variants are loss-of-function variants that cause protein truncation (54/62, 87.1%), with point mutations accounting for only 12.9% (8/62). The variants in HA20 patients with combined T1DM were nonsense or frameshift variants in the carboxy-terminal zinc finger (ZnF) coding regions or amino-terminal ovarian tumor (OUT) coding regions (**Table 2**).

The persistence of C-peptide secretion widely varies among patients after a long duration of type 1 diabetes (16). In the present case, even after 20 years, islet function has not completely failed (fasting C-peptide: 319.7 pmol/L). Unfortunately, data regarding changes in islet function after the onset of the disease were missing, and the reports of the remaining four patients in the literature did not present their islet functions; therefore, we could not summarize the characteristics of the process of islet function changes in HA20 combined with T1DM. However, if HA20 is speculated at the onset of the disease when the islet function is still good, early treatment using immunosuppressive or biological agents may limit or reverse the loss of beta cell function in T1DM with HA20. The clinical symptoms of our patients with HA20 were not serious and insulin administration provided adequate blood sugar control; therefore, we did not further try to use immunosuppressive or biological agents.

In this case, liver function revealed abnormally elevated transaminases, excluding other liver diseases. Recent research reported that patients with HA20 develop chronic liver involvement due to hepatic fibrosis, hepatocyte injury, and/or inflammatory T lymphocyte infiltrates with moderate NF-κB and/or NLRP3 staining (15). This indicates that HA20 may be present in patients who have multiple concurrent autoimmune diseases, Behcet’s-like symptoms, and unexplained liver function abnormalities.

In conclusion, we report a novel pathogenic mutation in TNFAIP3 that results in HA20 in a T1DM patient. We analyzed its clinical feathers and summarized five patients with HA20 in combination with T1DM. If T1DM is combined with other autoimmune diseases or clinical manifestations other than AITD, such as oral and/or genital ulcers and chronic liver injury, be aware of the possibility of HA20. Early diagnosis and timely treatment of HA20 may delay the progression of autoimmune diseases, including T1DM.

## Data availability statement

The original contributions presented in the study are publicly available. This data can be found here: https://figshare.com/articles/online_resource/0F2X000186_vcf/22873334.

## Ethics statement

The ethics committee approved the study of First Affiliated Hospital of China Medical University. The patients/participants provided their written informed consent to participate in this study. Written informed consent was obtained from the individual(s) for the publication of any potentially identifiable images or data included in this article.

## Author contributions

Study design: XW. Data collection: CC and XF. Manuscript drafting: CC and XF. Data interpreting: XW. Revision of the manuscript: XW. Approval of final version of the manuscript: CC, XF, and XW. All authors contributed to the article and approved the submitted version.
